# Prognostic value of post-induction chemotherapy ^18^F-FDG PET-CT in stage II/III non-small cell lung cancer before (chemo-) radiation

**DOI:** 10.1371/journal.pone.0222885

**Published:** 2019-10-11

**Authors:** Julien Ganem, Sebastien Thureau, Pierrick Gouel, Bernard Dubray, Mathieu Salaun, Edgar Texte, Pierre Vera

**Affiliations:** 1 Department of Nuclear Medicine, Henri Becquerel Cancer Centre and Rouen University Hospital, Rouen, France; 2 Department of Radiation Oncology and Medical Physics, Henri Becquerel Cancer Centre and Rouen University Hospital, Rouen, France; 3 QuantIF-LITIS, EA 4108-FR, CNRS, University of Rouen, Rouen, France; 4 Department of Pneumology, Rouen University Hospital, Rouen, France; Universite de Nantes, FRANCE

## Abstract

**Introduction:**

The purpose of our present study was to assess the prognostic impact of FDG PET-CT after induction chemotherapy for patients with inoperable non-small-cell lung cancer (NSCLC).

**Material and methods:**

This retrospective study included 50 patients with inoperable stage II/III NSCLC from January 2012 to July 2015. They were treated for curative intent with induction chemotherapy, followed by concomitant chemoradiation therapy or sequential radiation therapy. FDG PET-CT scans were acquired at initial staging (PET_1_) and after the last cycle of induction therapy (PET_2_). Five parameters were evaluated on both scans: SUVmax, SUVpeak, SUVmean, TLG, MTV, and their respective deltas. The prognostic value of each parameter for overall survival (OS) and progression-free survival (PFS) was evaluated with Cox proportional-hazards regression models.

**Results:**

Median follow-up was 19 months. PET_1_ parameters, clinical and histopathological data were not predictive of the outcome. TLG_2_ and ΔTLG were prognostic factors for OS. TLG_2_ was the only prognostic factor for PFS. For OS, log-rank test showed that there was a better prognosis for patients with TLG_2_< 69g (HR = 7.1, 95%CI 2.8–18, p = 0.002) and for patients with ΔTLG< -81% after induction therapy (HR = 3.8, 95%CI 1.5–9.6, p = 0.02). After 2 years, the survival rate was 89% for the patients with low TLG_2_ vs 52% for the others. We also evaluated a composite parameter considering both MTV_2_ and ΔSUVmax. Patients with MTV_2_> 23cc and ΔSUVmax> -55% had significantly shorter OS than the other patients (HR = 5.7, 95%CI 2.1–15.4, p< 0.01).

**Conclusion:**

Post-induction FDG PET might be an added value to assess the patients’ prognosis in inoperable stage II/III NSCLC. TLG, ΔTLG as well as the association of MTV and ΔSUVmax seemed to be valuable parameters, more accurate than clinical, pathological or pretherapeutic imaging data.

## Introduction

Non-small cell lung cancer (NSCLC) is one of the most frequent malignancies in Western countries and represents a leading cause of death by cancer [1]. If surgery is recommended in early stages, it is generally associated with radiation therapy and chemotherapy for patients with locally advanced disease [2]. Patients presenting with inoperable stage II/III NSCLC can benefit from induction chemotherapy before radiation therapy or before concomitant chemoradiation therapy.

Induction chemotherapy allows to start the treatment earlier, while preparing (chemo)-radiation therapy. It causes a reduction of tumoral volume and thus a narrowing of the fields of irradiation, which enables to reduce both volume and dose of radiation of the Organs at Risk (OAR), and to assess tumoral chemosensitivity of the primary tumor and nodal metastases.

Over the past few years, FDG PET-CT has proven its use for diagnosing [3], staging, evaluating tumor response [4] and has shown its potential as a prognostic imaging biomarker in lung cancer. Several studies have shown the prognostic implications of changes in standardized uptake value (SUV) and suggested that FDG PET-CT could predict the response to chemoradiation [5,6], induction chemotherapy [7] and radiation therapy alone [8,9]. A meta-analysis revealed that high tumoral uptake at staging could result in a worse prognosis, especially in early stages [10]. However, metabolic parameters in patients with NSCLC after induction therapy lead to controversial results in terms of prognostic evaluation [11,12]. Volume-based indices were useful for predicting therapy response after induction chemotherapy, however, that study concerned a majority of patients who underwent curative intent surgery [13]. The purpose of our present study is to assess the prognostic impact of FDG PET-CT after induction chemotherapy for patients with inoperable NSCLC.

## Material & methods

### Population and treatment

This retrospective study, approved by the institutional review board (approval number 1708B), included 50 patients from January 2012 to July 2015. Thirty-five of these patients were followed at the Henri Becquerel Cancer Centre whereas 15 of them were followed in other centres for a multi-centre trial (initiated by the Henri Becquerel Cancer Centre) in which patients with hypoxic tumoral areas could benefit from dose escalation radiotherapy (RTEP-5 NCT01576796)[14]. All these 50 patients’ clinical and imaging data were available and searchable in our centre. Patients provided written informed consent for their data and scans to be published anonymously.

Patients with inoperable stage II or III NSCLC, according to the 7^th^ edition of the International Union Against Cancer staging system, treated for curative intent with induction chemotherapy, followed by concomitant chemo-radiation therapy or sequential radiation therapy, were included. The 15 patients from the RTEP-5 study were included regardless of the hypoxic status of their tumor.

Patients who would have surgery after induction chemotherapy or presenting with metastases—at initial staging or after induction chemotherapy—were excluded.

Induction therapy consisted in 1 to 6 cycles of platinum-based chemotherapy. The following radiation therapy delivered 66 to 70 Gy in 33 to 35 daily fractions of 2 Gy, associated or not to concomitant chemotherapy.

All patients underwent FDG PET-CT scans at initial staging and before radiation therapy.

### PET-CT imaging

FDG PET-CT scans were acquired at initial staging (PET_1_) and between the end of the last cycle of induction therapy and the beginning of (chemo-) radiation therapy (PET_2_). For the 35 patients treated in Rouen, PET-CT scans were performed on a Biograph Sensation 16 Hi-Rez device (Siemens Medical Solutions, Erlangen, Germany, 29 patients), GE 710 (GE, 5 patients) and mCT 40 (Siemens, 1 patient). For the 15 remaining patients from the multi-centre trial, PET-CT scans were acquired on Gemini GXL (Philips, 2 patients), Biograph mCT 40 (Siemens, 5 patients), Biograph mCT (Siemens, 1 patient), Discovery ST (GE, 1 patient), Gemini TOF (Philips, 3 patients), Discovery ST 4 (GE, 1 patient), GE 690 (GE, 1 patient) and Biograph (Siemens, 1 patient). Patients were asked to fast for at least 6 hours before the time of ^18^F-FDG administration to ensure that the serum glucose and serum insulin levels were low. An activity of 3.5 to 5 MBq/kg of ^18^F-FDG was injected after 20 minutes of rest. Sixty minutes later (±10 min), the acquisition began with non-injected CT in the cephalocaudal direction. The images were acquired with the patients’ arms positioned over the head while breathing freely. The PET data were then acquired in the caudocephalic direction using a whole-body protocol (3 min per bed position). The delay between injection and acquisition was standardized to 60 minutes in order to obtain a normalized counting rate for all patients. Protocols of acquisition and reconstruction were inherent to each nuclear medicine department and the same for a given device. They followed EANM procedure guidelines [15].

### PET-CT analysis

PET_1_ and PET_2_ were analysed using a Planet Onco workstation (PlanetOnco, v.2.0; DOSISoft^®^). All lesions (primary tumor and involved lymph nodes) with significant uptake were considered, which allowed to determine 5 main parameters: SUVmax, SUVmean, SUVpeak (defined as the average SUV within a 1 cc spherical region of interest centred on a high uptake part of the tumor), metabolic tumor volume (MTV) using a 41% of SUVmax threshold and total lesion glycolysis (TLG), defined as the product of MTV by SUVmean. The response to induction therapy was assessed by calculating the deltas for each parameter, with:

$$\Delta_{\text{parameter}}=\left({\text{Parameter}}_{\text{PET}2}-{\text{Parameter}}_{\text{PET}1}\right)/{\text{Parameter}}_{\text{PET}1},\text{expressed}\;\text{in}\;\text{percent}.$$

In addition, CT data were also analysed with Telemis-Medical PACS interface (TM v.2.70) by measuring the summed lesions diameter as used in RECIST 1.1 [4].

### Patients follow-up and statistical analysis

The follow-up consisted in routine clinical evaluation and systematic imaging revaluation (FDG PET-CT or CT) at 3 and 12 months after the end of treatment, and then periodically. The follow-up duration was the time between the end of radiation therapy and the last day of clinical or imaging evaluation, or the date of death.

Time to progression was defined as the time between the end of radiation therapy and the date of local or metastatic recurrence assessed through any imaging modality.

Clinical data, such as performance status, age and weight loss, as well as histological subtypes and tumoral stage were collected. Statistical analyses were realized with MedCalc software. Predictive factors for overall survival (OS) and progression free survival (PFS) were analysed using univariate and multivariate analyses. Receiver operator characteristics curves were used to determine a cut-off value for parameters whose p-value was less than 0.05 in multivariate analysis. OS and PFS were graphically represented using the Kaplan-Meier method. A log-rank test was used to compare groups for each independent factor and a p-value< 0.05 was considered as a statistically significant difference.

## Results

Clinical data are summarized in Table 1.

**Table 1:** Population characteristics.

Patients	50
**Age (years)**	Mean : 63 (+/-9) Range : 37–84
**Sex:**	Number of patients
- M - F	44 6
**Histology:**	Number of patients
- Adenocarcinoma - Squamous cell carcinoma	28 22
**Tumoral stage:**	Number of patients
- IIA, IIB, IIIA - IIIB	23 27
**Induction chemotherapy (1 to 6 cycles):**	**Number of patients (N)**
- Platinum salt + Pemetrexed - Platinum salt + Gemcitabin - Platinum salt + Vinorelbin - Platinum salt + Taxanes	17 3 18 12
**Post- induction treatment:**	**N**
- Concomitant chemoradiation therapy - Radiation therapy	37 13
**Follow-up (months)**	Mean (+/- S.D.) : 21 (+/-11) Median : 19 Range : 2–45
**Events:**	**N**
- Relapse - Death	33 18

Fifty patients (44 men and 6 women) with a mean age of 63 years (+/- 9) were included in our study. Median follow-up was 19 months.

Twenty-eight (56%) of lung cancers were adenocarcinomas and 22 (44%) were squamous cell carcinomas.

Twenty-three cancers were staged as IIA, IIB or IIIA (46%) and 27 were staged as IIIB according to the UICC 7^th^ edition of TNM classification of malignant tumors (2009).

Induction therapy consisted in 1 to 6 cycles chemotherapy associating platinum salts to vinorelbin (36%), pemetrexed (34%), taxanes (24%) or gemcitabin (6%).

After induction chemotherapy, 37 patients (74%) were treated with concomitant radiochemotherapy and 13 with radiation therapy alone. Patients undergoing radiation therapy alone had more cycles of induction therapy than patients treated with concomitant chemoradiation (4.0 vs 2.3, p< 0.001).

During the follow-up duration, 66% of our population showed local or metastatic relapse and 18 patients (36%) died, mainly from disease progression or toxicities of therapy.

**Fig 1:**
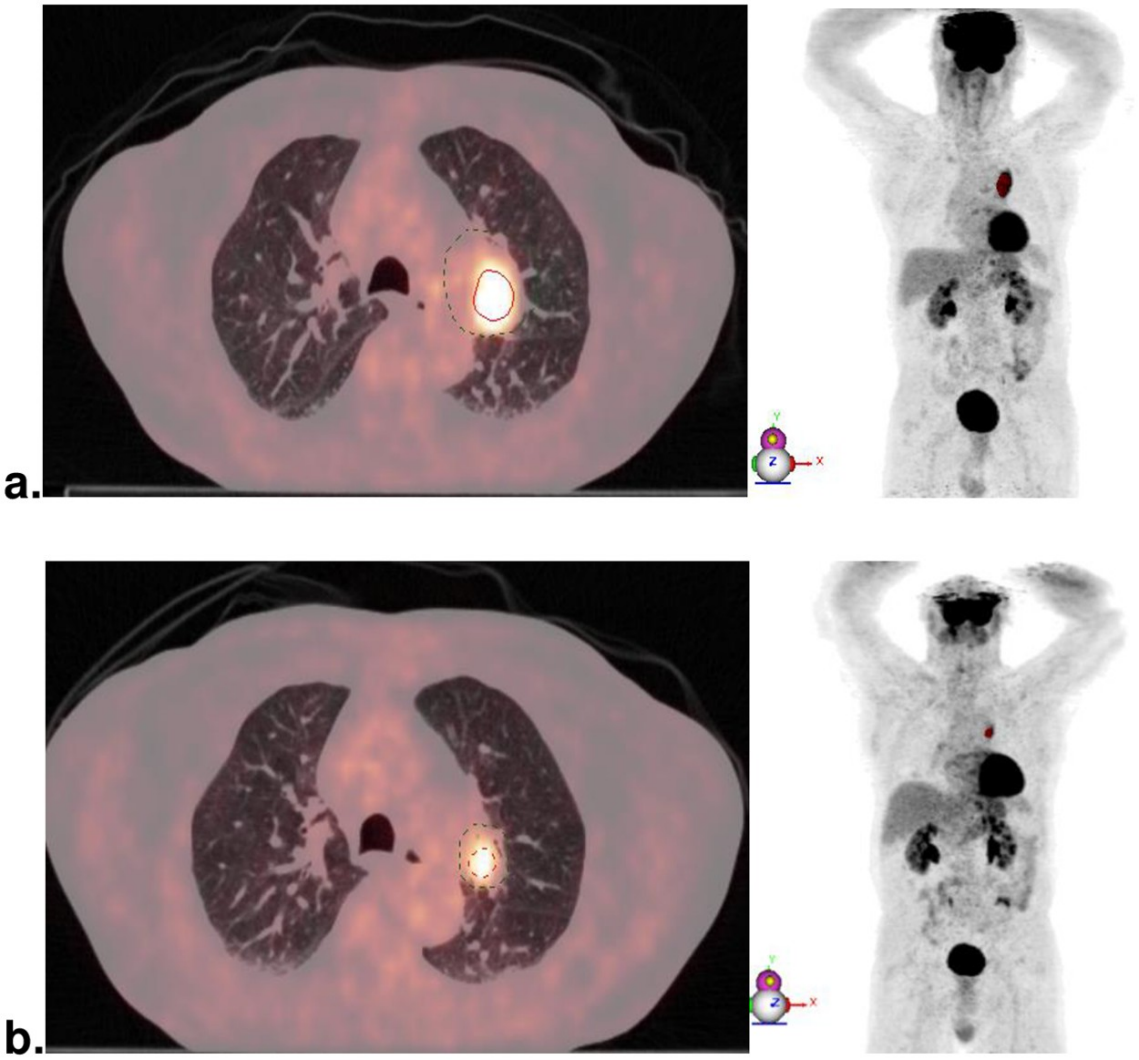
Tumoral response. 66-year-old patient with T_2_N_2_ (Stage IIIA) left upper lobe squamous cell carcinoma. At baseline **(a)**, tumor SUVmax was 12 g/ml, MTV 13 cc and TLG 89 g. After 2 cycles of carboplatin-paclitaxel **(b)**, SUVmax was 8.2 g/ml (ΔSUVmax = -33%), MTV 2.4 cc (ΔMTV = -82%) and TLG 11.3 g (ΔTLG = -87%). After 45 months, the patient was still alive and showed no sign of recurrence.

Fig 1 illustrates the changes between PET_1_ and PET_2_.

In univariate analysis, none of the following parameters was significant to predict the outcome of the population: performance status (p = 0.2), age (p = 0.38), histologic subtype (p = 0.86), pre-radiation weight loss (p = 0.07), the number of cycles of induction chemotherapy (p = 0.58), the association of concomitant chemotherapy during radiation (p = 0.85) or a locally advanced (IIIB) disease (p = 0.11).

Furthermore, lesion diameters used in RECIST 1.1 were measured using CT data to assess the response to the induction therapy. Twenty-six patients had a stable disease (52%), 23 showed a partial response (46%) and the remaining patient (2%) had a progressive disease. There was no statistically significant correlation between the response to induction chemotherapy and the outcome (for PFS: p = 0.08, for OS: p = 0.07).

Table 2 details information for each parameter of interest in PET_1_ and PET_2_, and the response to treatment with the delta of each parameter. Many parameters, mainly extracted from PET_2_ data, were significant to predict OS or PFS in univariate analysis. However, when considering overall survival, no parameter extracted from PET_1_ data was significant.

**Table 2:** PET data and univariate analysis.

	*Mean (+/- Standard Deviation)*	*Median*	*Range*	*PFS*	*OS*
***PET**_**1**_*					
*SUVmax_1_*	16 (+/-8)	14	4–50	p = 0.52	p = 0.79
*SUVmean_1_*	8 (+/-5)	7	2–31	p = 0.39	p = 0.54
*SUVpeak_1_*	14 (+/-7)	11	3–47	p = 0.53	p = 0.79
*MTV_1_*	73 (+/-65)	54	5–333	***p = 0*.*0004***	p = 0.75
*TLG_1_*	613 (+/-643)	399	28–3216	***p = 0*.*02***	p = 0.55
***PET**_**2**_*					
*SUVmax_2_*	9 (+/-9)	8	2–50	p = 0.31	***p = 0*.*004***
*SUVmean_2_*	5 (+/-5)	3	1–31	p = 0.46	***p = 0*.*01***
*SUVpeak_2_*	7 (+/-8)	6	0–47	p = 0.28	***p = 0*.*001***
*MTV_2_*	30 (+/- 28)	22	2–140	p = 0.056	***p = 0*.*03***
*TLG_2_*	160 (+/- 294)	75	3–1967	***p = 0*.*02***	***p< 0*.*001***
***Response***					
Δ*SUVmax*	-40% (+/- 31%)	-43%	_-_93%−_+_26%	p = 0.41	***p< 0*.*001***
Δ*SUVmean*	-43% (+/-30%)	-48%	_-_93%−_+_18%	p = 0.87	***p = 0*.*04***
Δ*SUVpeak*	-45% (+/- 31%)	-48%	_-_100%−_+_19%	p = 0.27	***p< 0*.*001***
Δ*MTV*	-45% (+/- 48%)	-54%	_-_94%−_+_164%	p = 0.85	p = 0.052
ΔT*LG*	-67% (+/- 36%)	-76%	_-_99%−_+_106%	p = 0.36	***p = 0*.*001***
Δ *Σ lesion diameters*	-28% (+/-22%)	-26%	_-_72%−_+_37%	p = 0.08	p = 0.07

In multivariate analysis (see Table 3), only TLG_2_ was an independent parameter for PFS, and both TLG_2_ and ΔTLG were independent factors for OS.

**Table 3:** Multivariate analysis.

	PFS	OS	Cut-off value	Sensitivity	Specificity	AUC
**TLG**_**1**_	p = 0.90					
**MTV**_**1**_	p = 0.06					
**SUVmax**_**2**_		p = 0.25				
**SUVmean**_**2**_		p = 0.42				
**SUVpeak**_**2**_		p = 0.15				
**MTV**_**2**_		p = 0.09				
**TLG**_**2**_	***p = 0*.*02***	***p = 0*.*02***	PFS: 97g OS: 69g	51.5% 88.9%	82.4% 59.4%	0.688 0.766
**ΔSUVmax**		p = 0.27				
**ΔSUVmean**		p = 0.63				
**ΔSUVpeak**		p = 0.84				
**ΔTLG**		***p = 0*.*03***	-81%	83%	56%	0.736
**Composite parameter**		***p = 0*.*01***	MTV_2_ < 23 cc or ΔSUVmax < -55% VS MTV_2_ > 23 cc and ΔSUVmax > -55%	72%	81%	0.771

Another parameter was analyzed: a composite considering both ΔSUVmax and MTV_2_ to isolate a subgroup of bad responders with low ΔSUVmax and high MTV_2_. P-values for this composite parameter were inferior to 0.05 in uni- and multivariate analysis, when considering overall survival. ROC curves analyses determined a threshold for each statistically significant parameter in order to separate the population into two groups.

When considering PFS, TLG_2_ was the only independent predictive parameter with a cut-off value of 97 g. A log-rank test revealed that there was a better prognosis for the 30 patients with low TLG_2_ (HR = 2.8, 95%CI 1.3–5.9, p = 0.002). After 12 months, 97% of the patients with low TLG_2_ showed no relapse versus 56% in the group of 20 patients with high TLG_2_. After 24 months, they were respectively 67% in the first group vs 34% in the second one.

**Fig 2:**
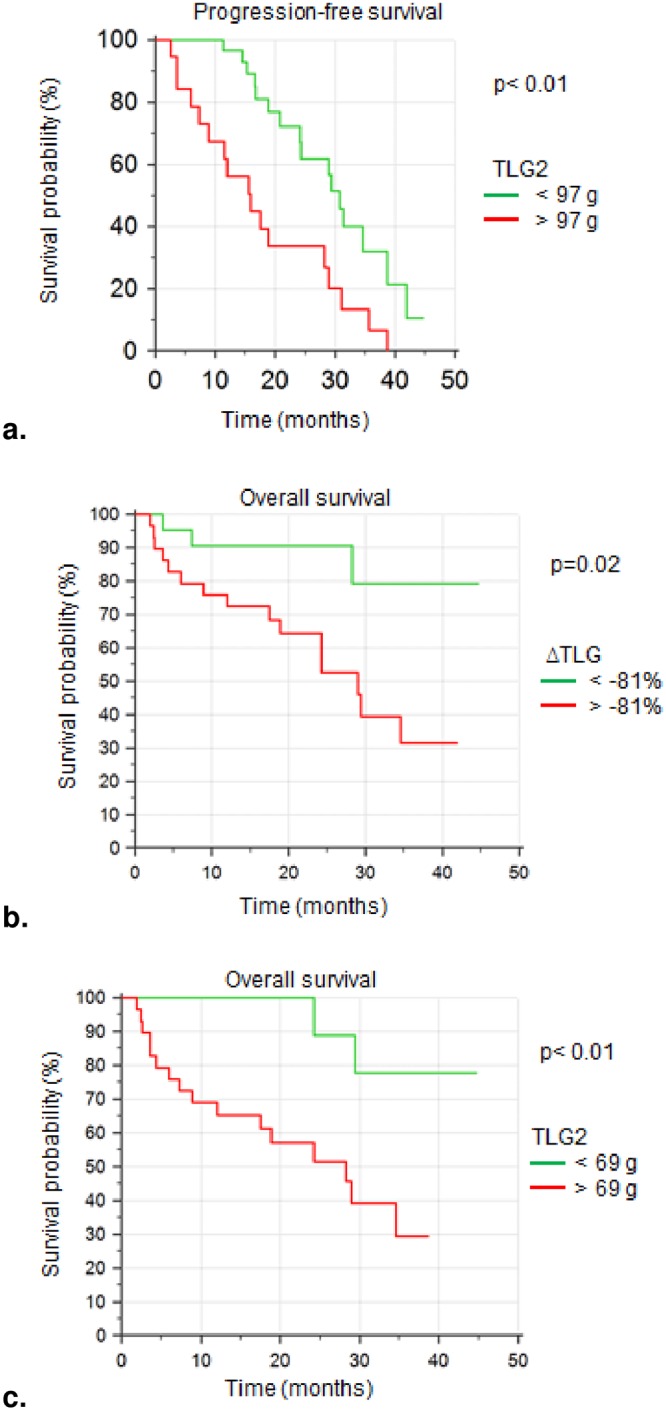
FDG PET parameters and survival curves. Survival curves using the Kaplan-Meier method. **(a)** PFS was longer for patients with low TLG_2_ (HR = 2.8, 95%CI 1.3–5.9, p< 0.01). After 2 years, the survival rate was 67% for the 30 patients with low TLG_2_ vs 34% for the 20 others. Median survival was respectively reached at 31 and 16 months. **(b)** 21 patients with high decrease of TLG after therapy (ΔTLG < -81%) showed longer OS (HR = 3.8, 95%CI 1.5–9.6, p = 0.02). After 1 year and after 2 years, survival rates for the 21 good responders were 91% versus 76% and 59% for the 29 bad responders. Median survival was not reached for good responders and was 29 months for the others. **(c)** Survival curves showed longer OS for patients with low TLG_2_ (HR = 7.1, 95%CI 2.8–18, p< 0.01). After 2 years, the survival rate was 89% for the 21 patients with low TLG_2_ (median survival not reached) vs 52% for the other 29 patients (median survival at 28 months).

For OS, ROC curves showed cut-off values of -81% for ΔTLG and 69 g for TLG_2_. A log-rank test showed that there was a better prognosis for patients with low TLG_2_ (HR = 7.1, 95%CI 2.8–18, p = 0.002) and for patients with a high decrease of TLG after induction therapy (HR = 3.8, 95%CI 1.5–9.6, p = 0.02). After 12 months, 91% of the 21 patients with high decrease of TLG were alive versus 76% of the remaining 29 patients, and 89% of the patients with low TLG_2_ survived versus 52% for the patients with high TLG_2_. Survival curves using the Kaplan-Meier method are represented in Fig 2.

**Fig 3:**
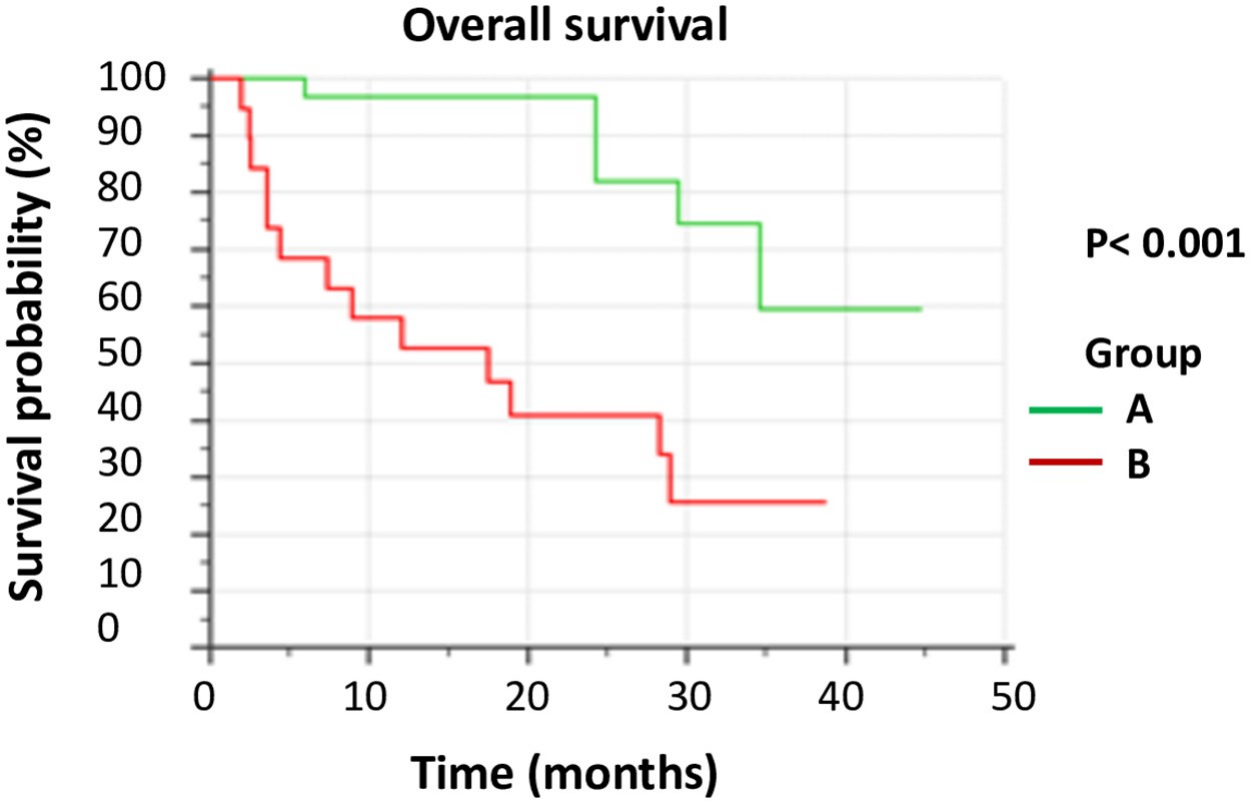
Overall survival using the composite parameter. Group A: 31 patients with low MTV_2_ or high decrease of SUVmax after induction therapy. Group B: 19 patients with high MTV_2_ (> 23 cc) and low decrease of SUVmax (ΔSUVmax> -55%). Median survival was not reached in Group A and was 17.5 months in Group B. HR = 5.7, 95%CI 2.1–15.4, p< 0.001.

We also used a composite parameter considering both ΔSUVmax and MTV_2_. Using the ROC curves, we determined a cut-off value for ΔSUVmax (-55%) and for MTV_2_ (23 cc). We were then able to isolate a subgroup of 19 patients with high MTV_2_ and low decrease of SUV after induction chemotherapy. When we compared the OS for this subgroup (Group B) to the OS of the 31 remaining patients (Group A), the log-rank test showed that OS was significantly longer for Group A vs Group B (HR = 5.7, 2.1–15.4, p< 0.001). After 12 months, survival rates were 97% for Group A vs 53% for Group B. After 24 months, they were 89% for Group A vs 41% for Group B (Fig 3).

## Discussion

We have shown that post-induction PET (PET_2_) could be an added value to assess the patients’ prognosis in inoperable stage II/III NSCLC. First, it allows the evaluation of response to induction chemotherapy, informs about the tumor chemosensitivity and about the estimation of the prognosis, thanks to the ΔTLG. Then, it seems that post-induction TLG (TLG_2_) is predictive of the outcome independently from pretherapeutic data.

In our study, data extracted from PET_2_ appeared to be more accurate than RECIST criteria or than clinical and histopathological data to assess the patients’ outcome.

We did not isolate any metabolic or volumetric parameter issued from pretherapeutic FDG PET (PET_1_) as a predictive factor for survival, in opposition to what is commonly found when reviewing the literature [10, 16–19]. Even though a few studies did not find any correlation between baseline uptake and survival [20], our hypothesis to explain these surprising results might be that our study considered a limited series of patients and could lack of power to show significant association between PET_1_ data and the population’s outcome. In that case, we can only assume that the parameters obtained with PET_2_ might predict the outcome with more reliability than those obtained with PET_1_. Of course, this should be studied in a prospective study with a larger cohort of patients.

The current standard for patients with locally advanced NSCLC includes a baseline FDG PET-CT followed by induction chemotherapy. A CT acquisition is then realized in position of treatment to prepare the radiotherapy which is to follow, concomitant or not to chemotherapy. Yet, this procedure only enables an assessment of morphologic tumor response, whereas PET-CT can determine both anatomic and metabolic tumor responses.

A recently published study showed that an early metabolic response using PERCIST 1.0 or EORTC criteria was more sensitive and accurate than with RECIST 1.1 criteria [21]. In addition, FDG PET may reveal and locate metastatic lesions, and identify patients with progressive disease. For instance, a phase II trial proposing dose escalation radiotherapy to ^18^F-MISO positive lesions in patients with NSCLC (RTEP-5 NCT01576796) showed that 9/79 patients (11%) were excluded because of metastatic progression on post-induction FDG PET [14]. These findings were also highlighted in one of the studies evaluating FDG PET after induction therapy, with 17% of patients with metastatic evolution after neoadjuvant therapy [22].

In addition to re-staging the disease and avoiding useless and potentially harmful treatment, PET_2_ could identify a population at high risk of relapse using prognostic parameters.

Among the tools assessed in our study, the composite parameter seems to be interesting to predict the outcome by isolating a subgroup of patients with the highest risk of progression.

Ho Yun Lee and S. M. Eschmann studies have used ΔSUVmax to distinguish between good responders to induction chemotherapy from bad responders [23,24]. In our study, the cut-off value for ΔSUVmax was -55%, in coherence to what was determined in those two studies (-50% and -60%, respectively).

Furthermore, post-induction volumetric indices were found to be prognostic tools regarding survival [13] or pathological response [25].

Once combined, ΔSUVmax and MTV_2_ became a significant tool in the multivariate analysis when considering overall survival. However, the use of this hybrid parameter has to be confirmed in a prospective or a cohort study.

A recently published study showed that ΔSUVmax was also an interesting parameter as well as ΔMTV, between baseline PET and interim PET after induction chemotherapy, to predict complete response after concurrent chemo-radiation therapy in patients with head and neck cancer [26].

Even though our results seem promising, our study suffers from a few limitations.

We retrospectively included patients who had benefited from curative-intent radiotherapy and undergone post-induction chemotherapy FDG PET. That limited the number of patients in our study since we did not include all the patients with inoperable NSCLC who had undergone neo-adjuvant chemotherapy. These facts have certainly impacted on the statistical power, which may have reduced the significance of other potential prognostic parameters. Moreover, we are not able to determine the amount of patients with progressive disease, since metastatic evolution or a too large tumor generally contra-indicate radiation therapy.

In addition, this retrospective recruitment lead to heterogeneity in induction chemotherapy, in both quantitative and qualitative aspects. However, the number of cycles of induction chemotherapy had no significant impact on the deltas (ΔSUVmax, ΔTLG, ΔSUVpeak, ΔSUVmean) or on the outcome.

Also, even though we did show that a locally advance disease (stage III B) was not a significant parameter to predict the outcome, we did not study if the involvement of lymph nodes was a valuable parameter to assess the patients’ prognosis because, since they had all been treated by induction chemotherapy because of an important tumoral mass, only four of them (8%) were N0 according to the TNM classification.

If a large prospective study confirmed the reliability of PET_2_ to identify a population at risk, we could imagine a change of treatment with a more personalized approach for these particular patients.

Several clinical trials are in process to evaluate the feasibility and the impact of adaptive therapy in NSCLC. For example, hypoxic lesions are known to be radio-resistant. Thus, escalation dose radiotherapy could be considered for these patients, in order to improve their survival. A phase II study of total dose increase in hypoxic lesions showed that dose escalation was feasible [14]. A survival analysis must then be conducted in a phase III clinical trial.

Furthermore, persistence of hypermetabolism in NSCLC during radiation therapy is highly predictive of relapse [27]. Currently ongoing trials study the impact on survival of escalation dose radiotherapy in these patients (RTEP7 NCT02473133, NCT01507428) [28,29].

Bad responders to chemotherapy could benefit from a switch to another line of chemotherapy as showed in a clinical trial of neoadjuvant chemotherapy [30]. Or, an alternative treatment associated to radiotherapy, such as immunotherapy [31,32], could be considered. Indeed, abscopal responses have been reported by physicians treating patients with non-small-cell lung cancer with ipilimumab combined with radiation [33].

The benefit of immunotherapy, such as anti-PD1 or anti-PD-L1 antibodies, in NSCLC will be assessed in ongoing or in future studies (NCT02768558, NCT0257843) [34,35]. In addition, a phase I trial has already evaluated the safety and tolerability of an immunocytokin (Selectikine) associated to radiotherapy [36].

The fact that PET_2_ potentially identified a subgroup of patients at high risk of relapse might lead to therapeutic prospective studies to improve their prognosis.

## Conclusion

Post-induction FDG PET might be an added value to assess the patients’ prognosis in inoperable stage II/III NSCLC. TLG and ΔTLG seemed to be valuable parameters and more accurate than clinical, pathological or pretherapeutic imaging data.

Moreover, the combination of volumetric and metabolic changes using a composite parameter (associating post-induction MTV and ΔSUVmax) seems to be an interesting tool to identify the patients with the highest risk of relapse after radiation therapy.

## Supporting information

S1 Dataset Minimal data set. (XLSX)
